# Practitioner Review: Clinical insights from attachment theory and research for professionals working with young children and their families

**DOI:** 10.1111/jcpp.70126

**Published:** 2026-02-25

**Authors:** Jessica E. Opie, Everett Waters, Robbie Duschinsky, Mårten Hammarlund, Sheri Madigan, Sarah Foster, Tommie Forslund, Ross Thompson, Howard Steele, Miriam Steele, Glenn I. Roisman, Ashley M. Groh, Peter Fonagy, Or Dagan, Alessandro Talia, Larissa Rossen, L. Alan Sroufe, Ed Tronick, R. M. Pasco Fearon, Pehr Granqvist, Abraham Sagi‐Schwartz, Alicia Lieberman, Elizabeth Carlson, Peter Zimmermann, Mary Dozier, Ashley Wazana, Jay Belsky, Phillip R. Shaver, Dante Cicchetti, Guy Bosmans, Carlo Schuengel, Karin Grossmann, Chantal Cyr, Karine Dubois‐Comtois, Marije Verhage, Anne Tharner, Mirjam Oosterman, Brian Allen, Judith A. Crowell, Pascal Vrtička, Matthew Woolgar, K. Lee Raby, Megan Galbally, Jeremy Holmes, Robert S. Marvin, Marinus H. van IJzendoorn, Marian J. Bakermans‐Kranenburg

**Affiliations:** ^1^ Department of Public Health and Primary Care University of Cambridge Cambridge UK; ^2^ Department of Psychology State University of New York at Stony Brook Stony Brook NY USA; ^3^ Department of Psychology Stockholm University Stockholm Sweden; ^4^ The Erica Foundation Stockholm Sweden; ^5^ Department of Psychology University of Calgary Calgary AB Canada; ^6^ Alberta Children's Hospital Research Institute University of Calgary Calgary AB Canada; ^7^ Department of Social Work, Education and Community Wellbeing Northumbria University Newcastle upon Tyne UK; ^8^ Department of Social Work Uppsala University Uppsala Sweden; ^9^ Department of Psychology University of California Davis CA USA; ^10^ Department of Psychology New School for Social Research New York NY USA; ^11^ Institute of Child Development University of Minnesota Minneapolis MN USA; ^12^ Department of Psychological Sciences University of Missouri Columbia MO USA; ^13^ Division of Psychology and Language Sciences University College London London UK; ^14^ Department of Behavioral Sciences Long Island University Brooklyn NY USA; ^15^ Institut de Psychologie, Faculty of Social and Political Sciences University of Lausanne Lausanne Switzerland; ^16^ Department of Counselling Psychology Trinity Western University Langley BC Canada; ^17^ Department of Pediatrics University of Massachusetts Chan Medical School Worcester MA USA; ^18^ Research Department of Clinical, Educational and Health Psychology University College London London UK; ^19^ The Anna Freud National Centre for Children and Families London UK; ^20^ Centre for Family Research University of Cambridge Cambridge UK; ^21^ Department of Psychology and the Center for the Study of Child Development University of Haifa Haifa Israel; ^22^ Department of Psychology Tel Hai College Tel Hai Israel; ^23^ Department of Psychiatry University of California San Francisco CA USA; ^24^ Department of Developmental Psychology University of Wuppertal Wuppertal Germany; ^25^ Psychological and Brain Sciences University of Delaware Newark DE USA; ^26^ Department of Psychiatry McGill University Montreal QC Canada; ^27^ Lady Davis Institute for Medical Research Jewish General Hospital Montreal QC Canada; ^28^ Centre for Child Development and Mental Health Jewish General Hospital Montreal QC Canada; ^29^ Human Ecology University of California Davis CA USA; ^30^ Clinical Psychology Research Group KU Leuven Leuven Belgium; ^31^ Section of Clinical Child and Family Studies Vrije Universiteit Amsterdam Amsterdam The Netherlands; ^32^ Department of Psychology University of Regensburg Regensburg Germany; ^33^ Department of Psychology Université du Québec à Montréal Montreal QC Canada; ^34^ Department of Psychology Université du Québec à Trois‐Rivières Trois‐Rivières QC Canada; ^35^ Center for the Protection of Children Penn State Children's Hospital Hershey PA USA; ^36^ Department of Psychiatry and Behavioral Health State University of New York at Stony Brook Stony Brook NY USA; ^37^ Centre for Brain Science, Department of Psychology University of Essex Colchester UK; ^38^ Department of Child and Adolescent Psychiatry Institute of Psychiatry, King's College London London UK; ^39^ National Adoption and Fostering Service, Michael Rutter Centre South London & Maudsley NHS Trust London UK; ^40^ Department of Psychology University of Utah Salt Lake City UT USA; ^41^ School of Clinical Sciences Monash University Melbourne VIC Australia; ^42^ Mental Health Program Monash Health Melbourne VIC Australia; ^43^ Department of Clinical Psychology University of Exeter Exeter UK; ^44^ School of Medicine University of Virginia Charlottesville VA USA; ^45^ The Ainsworth Attachment Clinic and Circle of Security Network Charlottesville VA USA; ^46^ Facultad de Psicología y Humanidades Universidad San Sebastián Sede Valdivia Chile; ^47^ Department of Psychiatry Monash University Melbourne VIC Australia; ^48^ William James Center for Research ISPA‐ University Institute of Psychological Lisbon Portugal

**Keywords:** Clinical translation, Early childhood, Parent–child relationships, Parenting, Attachment myths, Attachment‐informed interventions

## Abstract

Attachment theory, with its core concepts, perspectives, and insights developed over the past five decades, is influential for professionals working with young children. However, practitioners face challenges translating attachment theory and research into practical applications. This manifests in attachment myths, theoretical misinterpretations, and inconsistency of application. This state‐of‐the‐art review is authored by 47 attachment researchers and practitioners and examines key insights from attachment theory to facilitate attachment‐aware practice for professionals working with children and their caregivers. Following the ongoing debate on practical relevance in attachment theory, we present both ‘strict’ and ‘expansive’ translational perspectives on applications for addressing preventative or clinical attachment concerns. We first review core attachment propositions, based on replicated research of attachment and caregiving. We next address common misconceptions that hinder adequate practical applications. We present measures of attachment and sensitive parenting that might be helpful for practitioners. We also review evidence‐based and promising attachment interventions, discussing core components of (preventative) support for parents or caregivers and the children in their care. We emphasize that attachment theory's clinical value lies *not* in assigning attachment classifications, but rather in understanding crucial insights into caregiving and early socioemotional development (e.g., secure base phenomena; the value of safe, stable, and shared good‐enough care), developed in attachment research over the past 50 years, that may inform policy and clinical reasoning and areas for prevention and intervention.

## Introduction

Attachment theory has become highly influential in policy and clinical practice and is used widely by practitioners working with infants and young children, including psychiatrists, clinical psychologists, nurses, and social workers. Despite this widespread adoption, these professionals sometimes face challenges in translating attachment theory and research into practical applications. This review builds on an earlier landmark practitioner review (Zeanah, Berlin, & Boris, 2011) on the clinical applications of attachment theory for young children. It also updates a chapter in the Handbook of Attachment (Berlin, Zeanah, & Lieberman, 2016) on community‐oriented interventions, as we highlight meta‐analyses, interventions, and empirical studies published over the past decade. The current state‐of‐the‐art review is co‐authored by 47 experts from attachment research as well as from clinical practice in public health, clinical psychology, psychiatry, and counseling. Prioritizing best meta‐analytic evidence and results of randomized trials, we relied on the collective expertise of the authors from various disciplines and professions who agreed with the general findings and conclusions. The differentiation between a ‘strict’ and ‘expansive’ approach to translation of science to practice paved the way for finding common ground. Nevertheless, the current review is not a consensus or position statement of a task force outlining a protocol for diagnosis or treatment of attachment issues. By exploring progress and pitfalls in our field, we provide food for thought for both practitioners and researchers engaged with attachment theory and its potential applications. In Part I, we summarize generally endorsed key findings from attachment theory and research to provide guidance for policymakers and professionals working with children and their caregivers. In Part II, we explore attachment‐related measures with a focus on practical applications, and in Part III, we present some evidence‐based interventions or treatment of clinical concerns in the family context (e.g., harsh or punitive caregiving; caregiver stress or mental illness). Attachment‐specific resource recommendations for practitioners are also provided in Appendix S1. In the absence of consensus on translational approaches, in Parts II and III, we present ‘strict’ and ‘expansive’ translational perspectives on their usefulness for practitioners addressing preventative or clinical attachment concerns. Some readers might notice a faint echo of Paul Meehl's seminal work on ‘formal‐versus‐judgmental’ practical or clinical decision‐making (Meehl, 1967, p. 6).

## Part I: Understanding attachment and caregiving: core propositions and misconceptions

### Core attachment propositions

The relevance of attachment theory for practitioners lies in key propositions supported by decades of research and meta‐analytic evidence. We emphasize six key evidence‐based propositions that outline the current state of attachment theory. The core propositions might be used as a general framework for policy, preventative, and clinical practice. As we discuss below, the propositions have inspired attachment‐based parenting programs, and they might provide some directions for clinical observations and diagnosis.

- Attachment is a component of a relationship, not an individual's trait. It results from the child's disposition, in times of distress, to seek care and protection from and proximity to a stronger and wiser other who might provide a ‘safe haven’ for retreat during distress and a potentially ‘secure base’ from which the child can explore the environment.
- Attachment theory places significant emphasis on continuous and responsive care and limit setting during development, appropriately adapted to a child's development. This focus on caregiving guides practitioners to prioritize (preventative) interventions or placement decisions that enhance continuity of care.
- Attachment theory emphasizes that in the first 5 years, children develop expectations about safety, security, and trust within stable, close relationships through more or less consistent responsive interactions in continuous caregiving arrangements. These expectations develop into mental models (i.e., representations and secure‐base scripts) that promote (but do not determine) children's social competence in future relationships with peers, teachers, or partners.
- Attachment theory predicts, and research confirms, that secure attachment, wherein caregivers tend to consistently respond to infants' signals and needs, promotes later independence and self‐regulation, rather than dependency.
- Most children grow up in an ‘attachment network’ – comprising caregivers such as parents, grandparents, and childcare workers – who may provide secure bases and safe havens in the children's everyday life. Children may develop different attachment relationships with different caregivers, and current evidence suggests that the attachment relationship with mothers is, in itself, not more important for developmental outcomes than with fathers.
- Insecure or disorganized attachments cannot be identified with later (developmental or attachment) disorders or clinical problems; they can only predict an elevated chance of problems ahead if they concur with other risks at the social or individual level.

The core propositions are strictly probabilistic, implying that predictions from those propositions only support group‐level associations with many individual exceptions to the rule. Thus, the core propositions cannot be used to predict with certainty the future development of an individual child. The practitioner's ‘expertise‐by‐experience’ remains indispensable, but the core propositions might serve as touchstones for validating practical decisions or interventions. In clinical contexts, valid attachment assessments with sufficient sensitivity and specificity are not yet available, and clinicians have to rely on their sharp clinical eye and extensive experience‐based judgment. Clinicians with a thorough understanding of attachment theory, its concepts, and core findings may feel confirmed in their experience. They are able to effectively derive confirmation and reassurance from attachment theory and research with awareness of the limitations. They remain open to alternative interpretations and avoid definitive conclusions, conscious of attachment theory as work‐in‐progress. They clearly distinguish their clinical impressions from so‐called gold‐standard attachment assessments or measures validated for group‐level use instead of clinical case‐level application.

### Attachment misconceptions

Attachment theory has often been misread. As Foster et al. (2025) observe: ‘Students, practitioners, and the families they work with are likely to encounter misinformation about attachment from various sources. This misinformation is widely available, can take the form of eye‐catching claims, and much of it sounds plausible and promises ready practice‐relevance’ (p. 3). Additionally, theorists, practitioners, and even parents have projected their own understandings and personal perspectives onto the theory. Such misconceptions have led some to overly simplified and deterministic interpretations of attachment theory's central propositions, as highlighted by attachment researchers (Duschinsky et al., 2021; Hammarlund, Granqvist, Elfvik, Andram, & Forslund, 2022). While there are points of both agreement and disagreement between attachment researchers and practitioners, we suspect that these misunderstandings have contributed to challenges in the accurate translation of attachment research to practice, with the risk of a breakdown in dialog between researchers and practitioners (Beckwith et al., 2022). Dispelling the misunderstandings, busting myths, and clarifying any idiosyncratic interpretations of attachment theory would significantly benefit clinicians and, in turn, help clinical discussions contribute effectively to the interpretation of research findings and to new research (Fearon, 2019; Van IJzendoorn & Bakermans‐Kranenburg, 2024).

Based on research and clinical experience, we identify and address some key ‘myths of attachment’ – misconceptions that make attachment theory difficult to communicate and apply in community and clinical settings. Understanding attachment theory misconceptions is important for practitioners, as these misconceptions can have harmful consequences, manifesting across multiple policy and practice domains. We report on potential costs associated with the adoption of each myth in clinical practice or parenting support and refer to current theory and best empirical evidence to redress them.

**Misconception 1: The core insight of attachment theory is the classification of a child's attachments into Secure, Avoidant, Resistant, and Disorganized.** This notion plays a central role in much popular literature on attachment theory. However, clinically as well as in other contexts, overemphasizing attachment classifications leads to trait and typological thinking. Focusing on classification can overshadow more preventative or therapeutically relevant aspects of attachment theory, such as the caregivers serving as a secure base and a safe haven. Further, emphasizing classification can lead to stigmatizing labels, leading to self‐fulfilling prophecies. Although classifications are valuable research tools, they are primarily useful when conducting population studies to understand group trends (Forslund et al., 2022). They are not (currently) validated or intended to be used clinically at the individual level. Instead of attempting to categorize relationships, practitioners may focus on supporting caregivers to sensitively respond to children's needs and promote secure base behavior.
**Misconception 2: Attachments can be strong or weak, meaning that a child can be ‘over‐attached’ or ‘under‐attached’.** This incorrect perspective shifts the focus from attachment quality (e.g., secure vs. insecure) to a quantitative view of ‘how much’ attachment exists. Security is about the quality of the relationship and the child's confidence in the caregiver's availability (Ainsworth, Blehar, Waters, & Wall, 1978/2015). Attachment relationships differ in the extent to which the infant or child is confident in the caregiver's availability and responsiveness, and their ‘being there for me’ as a secure base and a safe haven, and do not reflect differences in the strength of the attachment relationship.
**Misconception 3: Once established, attachment (in)security remains unchanged throughout the life course.** Contrasting with this idea, research shows that attachment classifications can change throughout life due to new experiences, relationships, or therapeutic interventions. Although early attachments are influential, childhood attachments are by no means fixed (Booth‐LaForce & Roisman, 2021; Opie et al., 2021). The misconception of stable attachment (in)security can result in caregiver guilt, anxiety, and self‐blame about suboptimal caregiver quality in the first few years of the child's life. It may also undervalue the efficacy of attachment‐informed interventions or therapeutic efforts.
**Misconception 4: Attachment classifications can be reliably deduced from a child's behavior in the clinicians' waiting room.** This misconception bypasses validated assessment protocols requiring specific procedures and trained observers and that are meant for comparisons at the group level (e.g., Ainsworth et al., 1978/2015; Waters & Deane, 1985). Other uses may lead to premature clinical judgments. Clinical observations of the child, enriched with attachment concepts and insights, can be clinically helpful without assessing attachment classification. Brief observations in a single context, such as a waiting room, have not been demonstrated to capture the complexity of attachment relationships, and making informal assessments goes against professional standards for attachment classification.
**Misconception 5: Only secure attachment is adaptive, and insecure attachment is pathological, requiring assessment and intervention.** This perpetuates the false belief that insecure infants are necessarily unhappy, developmentally compromised, or at significant risk – when insecure attachment classifications actually can represent adaptive strategies to cope with a specific context and do not necessarily predict negative outcomes. It also increases the risk that parents are considered ‘bad’ when children are insecurely attached. Consequently, in child welfare, perceived insecure child attachment is sometimes cited as a factor contributing to placement decisions. Insecure classifications, present in roughly half of children, help maintain caregiver availability within their relationship context. Research supports only small‐to‐moderate associations between attachment classification and developmental outcomes (Groh, Fearon, Van IJzendoorn, Bakermans‐Kranenburg, & Roisman, 2017).
**Misconception 6: Disorganized attachment is an indicator of child maltreatment.** This misconception may result in unnecessary out‐of‐home child placement for those identified as disorganized, who are seen as needing protection from maltreatment. Disorganized attachment can develop through multiple pathways (e.g., social disadvantage, caregiver mental health difficulties, family stress, caregiver's unresolved trauma, or maltreatment; Cyr, Euser, Bakermans‐Kranenburg, & Van IJzendoorn, 2010). A more accurate understanding recognizes that although maltreatment can contribute to disorganized attachment, it is just one of many potential pathways. Not all maltreated children develop disorganized attachment, and not all children with disorganized attachment have experienced maltreatment (Forslund et al., 2022).
**Misconception 7: Insecure or disorganized attachment is equivalent to attachment disorder.** This idea can create conceptual confusion in clinical reports and may result in the overidentification of insecurely attached children as attachment disordered. Unlike the two attachment disorders described in the ICD‐11 and DSM‐5‐TR (i.e., Reactive Attachment Disorder [RAD] and Disinhibited Social Engagement Disorder [DSED]), insecure or disorganized attachment is not a formal diagnosis. Attachment insecurity and disorganized attachment relate to the developmental perspective that examines typical attachment classifications across typical populations. In contrast, the diagnostic perspective focuses on identifying clinically significant attachment disorders in vulnerable populations. Disorganized attachment is present in less than 20% of young child‐caregiver dyads and is part of typical individual differences among populations, while attachment disorders represent clinical psychopathology and are rare, primarily observed in (formerly) institutionalized children (Bakermans‐Kranenburg et al., 2011; Zeanah et al., 2011).
**Misconception 8: The neural substrates of attachment are localized in ‘primitive’, ‘reptilian’, or ‘mammalian’ brain areas predominantly in the ‘right brain’.** This misconception has led to the suggestion that attachment interventions necessitate ‘right brain‐to‐right brain communication’ between therapists and clients, and other ‘neuroscientific’ applications. Current social neuroscience accounts of human attachment show that the complex attachment system is orchestrated by several extended and interconnected neural networks spanning both hemispheres and involving many cortical and subcortical brain areas (White, Kungl, & Vrticka, 2023). Neuroscientific attachment research has just started to try to unravel the role of brain structures and function in attachment and parenting, and unfortunately it still is too early for evidence‐based practical applications (Van IJzendoorn & Bakermans‐Kranenburg, 2024, ch. 10).
**Misconception 9: ‘Attachment therapies’ such as ‘rebirthing’, ‘holding therapy’, and ‘re‐parenting’ are evidence‐based, attachment‐informed interventions.** This misconception is likely due to widespread advertising of ‘attachment therapies’ as equivalent to ‘attachment interventions’. Such ‘attachment therapies’ can, however, be physically and psychologically harmful to children, lack scientific evidence, and have even resulted in death (American Academy of Child and Adolescent Psychiatry, 2008; Zeanah et al., 2011). In contrast to evidence‐based attachment‐informed interventions, ‘attachment therapies’ use harmful techniques, such as extremely insensitive physical restraint. Evidence‐based attachment interventions aim at enhancing caregiver sensitivity primarily through promoting caregivers' reflection on their real‐life interactions with the child and exploring change of their parenting behaviors in a safe, nonjudgmental context for themselves as well as for their child.

## Part II: The role of attachment measures

### Strict and expansive translational perspectives on applying attachment measures

In attachment theory and research two hotly debated diverging lines of reasoning on applying attachment measures to practice are apparent, of course with all kinds of modalities in between. In the line of ‘strict’ translational reasoning, the evidence‐based limitations of what attachment measures can offer to practitioners are emphasized. Attachment measures, including measures to assess attachment‐relevant parenting, have been developed and validated in large samples in the context of attachment research. Popular measures used in attachment research often have complex scoring systems and would require substantial time for training to be used in a reliable and valid way. But more importantly from this perspective, even if practitioners are trained to become reliable coders, still a lot of work on specificity and sensitivity has to be done to make attachment measures feasible for screening or application on single cases for diagnostic purposes, for example, in court proceedings or custody evaluations asking for individual decision‐making (Forslund et al., 2022). Currently, attachment measures show too many false positives or false negatives, even for screening purposes to isolate families at risk who might be prioritized to receive focused, supportive interventions (Van IJzendoorn, Bakermans, Steele, & Granqvist, 2018). The utility of attachment classifications for understanding the needs of individual children is unproven, and the current attachment measures remain unsuitable to decide about preventative or therapeutic courses of action (Hammarlund et al., 2022). For all kinds of reasons, the attachment research community has in this respect not yet invested sufficient resources in translation of research to practice to be of any genuine help to experienced practitioners (Galbally, Stein, Ambrosius Hoegfeldt, & Van IJzendoorn, 2020; Schuengel, Verhage, & Duschinsky, 2021; Van IJzendoorn & Bakermans‐Kranenburg, 2021).

The alternative line of ‘expansive’ translational reasoning emphasizes that while research contexts generally demand formal attachment scoring, clinical practice may benefit from a more flexible appraisal approach. Measures developed through research on attachment – without the need for formal procedures, scoring, or coding – may help to sharpen the lens through which the complex clinical reality of parents and children struggling with attachment issues is surveyed. Many clinical psychologists and psychiatrists have participated in workshops to be trained in the use of attachment‐related measures without becoming reliable coders, but have nevertheless experienced considerable advantages for their capacity to identify and work with attachment‐related issues in clinical practice. Attachment and parenting measures may help practitioners to focus better on relevant attachment behaviors in various developmental stages and contexts. The originator of attachment theory, John Bowlby, viewed attachment through the lens of behavioral biology (ethology), with its emphasis on detailed observation of the content, organization, and function of behavior in naturalistic settings (Tinbergen, 1963). Unfortunately, informal use of attachment and sensitivity measures lacks the psychometric validity demonstrated for the formal, standardized assessments. However, for practitioners, the alternative would be to rely on transient impressions or anecdotal observations, information from preschool teachers or medical records, or other non‐validated assessment tools. Thus, although information from informally applied attachment measures should always be used with recognition of constraints, and interpreted taking the departure from standardized conditions into account, such assessments may provide clinical observations with some structure and an overarching theoretical framework.

From both the strict and expansive translational perspectives, the use of core concepts and propositions of attachment theory in policy and (clinical) practice is advocated. Importantly, from both approaches, building on more general knowledge embedded in the core propositions mentioned above is needed in order to ask the right questions about the children's needs for safe, stable, and shared care and how their caregivers might be enabled to meet those needs. For young children, being able to achieve comfort and protection in times of distress, and to explore and engage with the gradually expanding world around them, is of crucial developmental importance. In addition, the caregivers' capacity to sensitively set boundaries in a manner that facilitates children's ability to function in a gradually more important and complex social world outside of the family of origin is important. From such core attachment findings, important questions for understanding the problems of parents, caregivers, and their children can be gleaned, and observations addressing these questions can inform clinical hypotheses as well as possible decisions about how to support the individual child and caregivers:

1. How and to what extent does the child seek proximity to the caregiver in times of distress?
2. How does the caregiver respond to the child's distress signals?
3. Does the caregivers' response to the child seem to help them to regulate their emotions?
4. To what extent does the child seem free to explore and engage with the social and physical surroundings?
5. Are there aspects of the interaction that seem to frighten or alarm the child?
6. What feelings and thoughts does the child seem to have about the self in relation to the caregiver, and how may these relate to the child's attachment behavior?
7. How do the caregiver and the child handle relational friction or conflict?
8. How does the caregiver interpret the child's behavior in such situations?
9. How do these situations and interactions resonate or contrast with the caregivers' own attachment history?

From a *strict* translational view, practitioners interested in working from an attachment perspective typically would do well to focus on such questions rather than on specific attachment measures that have documented evidence only for research purposes in large samples. However, recent developments might be positioned to create psychometrically responsible adaptations of existing attachment and sensitivity measures for use in screening, diagnosis, or treatment of individual cases to enhance preventative and clinical effectiveness (Cooke, Eirich, Racine, Lyons‐Ruth, & Madigan, 2020; Cyr, Dubois‐Comtois, Paquette, Lopez, & Bigras, 2022). Madigan et al. (2024) has initiated a Caregiving and Attachment Research and Education collaboration (CARE; https://carecollaboration.org/) with the aim of bridging the gap between research and practice. In time, therefore, attachment measures may become available that would be considered appropriate for use in practice, also from the strict translational view.

From an *expansive* translational perspective, several attachment and parenting measures are relevant to assist practitioners in understanding caregivers and the children in their care better, even without a demanding scientific evidence base for their use outside of research settings. Here we list the most promising ones for expansive translational purposes, with some clinical notes.

### Examples of attachment measures

#### 1. Child attachment behavior

##### Strange Situation Procedure (SSP)

The Strange Situation Procedure (SSP; Ainsworth et al., 1978/2015) is a structured observational assessment of infant and toddler attachment, with two brief separations from the caregiver and interactions with a stranger, conducted in a novel environment. This observational procedure activates the child's attachment system and examines children's *attachment‐exploration balance*, revealing how a child uses the caregiver as a potential secure base and safe haven. Within the SSP, the moments of child‐caregiver *reunion* (not separation) prove particularly informative, with differences between infants who can effectively use their caregivers as a secure base and regulating resource and those who cannot. Securely attached children strike the balance between seeking comfort when distressed (e.g., due to the separation) and exploring the world, resuming play when comforted upon reunion; this balance between exploration and proximity‐seeking is what Ainsworth called the *secure base phenomenon*. Insecure‐avoidant attachment behavior is characterized by avoidance of the caregiver upon reunion, with a focus on (superficial) *exploration* rather than proximity or contact. Children with an insecure‐avoidant attachment relationship seem independent and unaffected by the separation from the caregiver, yet behavioral and physiological measures indicate distress. Insecure‐resistant children engage in attachment behavior when exploration might be expected, maximizing the attentional focus on the caregiver. Children with a disorganized attachment relationship may show mostly the same behaviors as children with secure, avoidant, and resistant relationships, but with moments of disruption that do not fit these three patterns. They may seem fearful, display contradictory behaviors or behaviors that lack behavioral organization (e.g., stilling and freezing).

###### Clinical relevance

SSP administration and scoring require extensive training. It is important to realize that key child behaviors, such as avoidance in the SSP, can be quite different from behaviors at home. The behaviors that emerge within the SSP should be interpreted as relationship‐specific patterns triggered within the particular context and period of the child's life. From a strict translational perspective, there is no convincing evidence that using the SSP promotes better treatments or clinical decision‐making. From an expansive translational perspective, learning to administer and score the SSP can help familiarize practitioners with the meaning of secure base behavior.

##### Attachment Q‐Set (AQS)

The AQS (Vaughn, Waters, & Teti, 2021) provides researchers with a naturalistic observational tool for assessing attachment security in young children. After on average 120 min of observation, the 90 behavioral AQS items are sorted from most to least descriptive of the child in a forced distribution of nine piles with 10 items each. The conceptual contribution of AQS lies in focusing attention on the child's behaviors, suggesting confidence or lack of confidence in secure base and safe haven availability, which is the heart of attachment theory. By observing child behaviors during everyday interactions, researchers evaluate how effectively children use their caregiver as a secure base for exploration. The AQS conveys a sense of (in‐)secure attachment relationships in ordinary contexts without requiring stressful separation procedures as is the case with the SSP.

###### Clinical relevance

The AQS provides a method to systematically observe attachment in the natural context, enabling repeated assessments over a broader age range than the SSP. From a strict translational perspective, these features make the AQS very useful in the context of (randomized) intervention studies. From an expansive translational perspective, familiarity with the AQS items may enhance practitioners' observational skills and provide a framework for discussing attachment behaviors with caregivers. It may also help them notice significant behaviors occurring during clinical sessions. The AQS may allow for a more nuanced understanding of the child's relational patterns and help target interventions to strengthen specific aspects of the attachment relationship.

#### 2. Attachment narratives

Narratives provide some insight into children's or caregivers' beliefs, expectations, perceptions, appraisals, and wishes in relation to current or previous attachment experiences. Examples of attachment narrative tasks eliciting these narratives include the Attachment Script Assessment (Waters & Rodrigues‐Doolabh, 2004; Waters & Waters, 2006) and the Adult Attachment Interview (George, Kaplan, & Main, 1985).

##### Attachment Script Assessment (ASA)

Participants generate brief narratives (75–300 words) using prompt‐word sets that imply secure base interactions. Based on the individual's history of repeated attachment experiences, these prompts activate the script, influencing story production. Elements of a secure base script include: (1) reciprocal, positive interactions between attachment partners, (2) clear signaling of attachment needs to relationship partners when problems arise, (3) provision of emotional and physical support by the attachment figure that effectively resolves the partner's problem, and (4) resumption of reciprocal, positive interaction or constructive engagement with the environment. Thus, knowledge of a secure base script in narratives generated during the ASA is meant to parallel secure base behavior exhibited in the lab environment during the SSP and during naturalistic home observations as evaluated via the AQS (Waters & Waters, 2021).

###### Clinical relevance

Rooted in the working model concept, the ASA (Waters & Waters, 2006) can be used in middle childhood, adolescence, and adulthood to evaluate the degree to which individuals have knowledge of, and access to, a secure base script derived from early attachment experiences. From a strict translational perspective, the instrument thus bridges the gap between infant and adult measures of attachment and enables longitudinal studies of attachment development in typical and atypical groups. Promising research evidence relates differences in secure base script knowledge to variance in interactions with attachment figures or (in the case of caregivers) their children. From an expansive translational perspective, the ASA may clarify for practitioners what is meant by a secure base script and how attachment interactions typically unfold. This insight can be used by practitioners to identify misinterpretations regarding attachment signaling and appropriate support provision. The ASA can then be used in whole or in part, or supplemented with practitioners' own questions and probes. Given the dyadic nature of secure‐base interactions, it could be beneficial to conduct child assessments in conjunction with their caregivers' narratives.

##### Adult Attachment Interview (AAI)

The AAI is a semi‐structured interview that addresses parents' current representations of their own childhood attachment experiences; its original aim was to predict how the attachment relationship with their offspring would unfold in the first few years of life (George et al., 1985). From the AAI, several rating scales and an overall classification are derived: (1) secure or autonomous interviews reflecting coherent integration of caregiver experiences (positive *and* negative), and an internalized secure base for exploring attachment‐related thoughts and feelings; (2) insecure‐dismissing interviews, reflecting idealization or devaluation of attachment experiences, and deactivation of attachment distress; (3) insecure‐preoccupied interviews, reflecting enmeshment with caregivers, anger (heightened activation of attachment distress), or limited autonomy; and (4) interviews reflective of enduring unresolved grief or trauma during discussions of experiences of loss or abuse from attachment figures (Bakermans‐Kranenburg, Dagan, Cárcamo, & Van IJzendoorn, 2025; Hesse, 2016).

###### Clinical relevance

The AAIs of caregivers predict child attachment security with a modest but robust effect size (Madigan et al., 2024). From a strict translational perspective, little evidence is available for differential parenting intervention effectiveness in caregivers with divergent attachment representations (Bakermans‐Kranenburg, Juffer, & Van IJzendoorn, 1998; Cassibba, Castoro, Costantino, Sette, & Van IJzendoorn, 2015; Tyrrell, Dozier, Teague, & Fallot, 1999). From an expansive translational point of view, the documented importance of *the way* in which interviewees talk about their past attachment experiences is relevant. The AAI may reveal defensive processes, misapprehensions, and attributions, providing a window into how individuals manage emotional distress. Additionally, it permits the observation of reflective functioning (i.e., caregiver's ability to consider other people's mental states when interpreting their child's behavior; Fonagy, Steele, & Steele, 1991; Steele & Steele, 2008). Moreover, the AAI can be valuable for uncovering unresolved trauma or loss that might impact caregiving behavior and affect the therapeutic relationship (Steele & Steele, 2008). Bowlby (1949) speculated that practitioners wishing to help worried caregivers and their emotionally troubled children might recognize the value of hearing caregivers about their own attachment histories and current perspective on their family of origin.

#### 3. Parenting measures

When attachment or parenting concerns arise, direct observations of caregiver behaviors may often provide more valuable and targeted information than measures focused on attachment in a narrow sense. Caregiver difficulties have been associated with troubling caregiving markers (Madigan et al., 2006). In contrast, caregiver sensitivity is predictive of the development of secure attachment (Madigan et al., 2024).

##### Maternal Sensitivity Scales

Caregiver sensitivity is characterized by prompt, appropriate, and contingent responses to infant signals, delivered with consistency, accessibility, and respect for the young child's budding autonomy (Ainsworth et al., 1978/2015). Ainsworth (1969) developed the path‐breaking approach to assessing infant‐caregiver interaction with the Maternal Sensitivity Scales that comprises four 9‐point scales: (1) Sensitivity vs. Insensitivity to child's signals; (2) Cooperation vs. Interference with child's ongoing behavior; (3) Physical and Psychological Availability vs. Ignoring and Neglecting; and (4) Acceptance vs. Rejection of child's needs (see Posada, Waters, Vaughn, Pederson, & Moran, 2021). These scales provide key insights into attachment relationships and predict long‐term developmental outcomes in a robust and possibly stronger way than attachment measures proper (Deneault et al., 2023). Derivatives of the Maternal Sensitivity Scales include the Emotional Availability Scales (Biringen, Derscheid, Vliegen, Closson, & Easterbrooks, 2014), Erickson's rating scales for Supportive presence and Intrusiveness (Egeland, Erickson, Clemenhagen‐Moon, Hiester, & Korfmacher, 1990), the Maternal Behavior Q‐Sort (Moran, Pederson, & Bento, 2009), and the Ambiance (Lyons‐Ruth, Bronfman, & Parsons, 1999).

###### Clinical relevance

From a strict translational perspective, Ainsworth's observational measure is relevant because the scales have often been used as an outcome of treatment in randomized controlled trials of attachment‐based parenting interventions (e.g., Van IJzendoorn, Schuengel, Wang, & Bakermans‐Kranenburg, 2023) and show predictive power for child‐caregiver attachment quality in meta‐analyses (e.g., Madigan et al., 2024). According to the strict translational perspective, the scales are still in need of replicated empirical evidence for screening purposes or in clinical practice. From an expansive translational perspective, however, the scales may be used by practitioners to set treatment aims and target insensitive parent behaviors, for instance, using the Ambiance‐Brief (Cooke et al., 2020).

## Part III: Attachment–informed interventions

Attachment–informed interventions have the shared goal of supporting caregivers to enhance their sensitivity when interacting with their child and increase the chance for secure child‐caregiver attachment. They further aim to promote family preservation and decrease the risk for child out‐of‐home placement and, hence, separations from and losses of attachment figures. The ultimate goal of this approach is to provide vulnerable children with the safe, stable, and shared (Triple S) family‐based care they require (Forslund et al., 2022; Van IJzendoorn & Bakermans‐Kranenburg, 2024).

We describe two programs with strong evidence from multiple randomized control trials (RCTs): Attachment and Biobehavioral Catch‐up (ABC; Dozier, Roben, Caron, Hoye, & Bernard, 2018) and Video‐feedback Intervention to promote Positive Parenting and Sensitive Discipline (VIPP‐SD; Juffer, Bakermans‐Kranenburg, & Van IJzendoorn, 2017). We also review five promising programs with smaller evidence bases: Attachment Video‐feedback Intervention (AVI; Moss et al., 2011), Child–Parent Psychotherapy (CPP; Lieberman, van Horn, & Ghosh Ippen, 2005), Circle of Security (COS; Cooper, Hoffman, & Powell, 2009; Powell, Cooper, Hoffman, & Marvin, 2013), Group Attachment‐Based Intervention (GABI; Steele, Murphy, Bonuck, Meissner, & Steele, 2019), and Minding The Baby (MTB; Slade, Sadler, Eaves, & Webb, 2023). The strict translational approach would argue that the fidelity of protocol implementation of evidence‐based programs is key, because effectiveness otherwise is not indicated. For most programs, training and certification are required. The expansive translational approach encourages (also) the use of program ingredients to explore what works best in a specific case or group setting. From this perspective, only part of a program might be implemented, mixed with parts of other programs, whatever their evidence base is, and fidelity is not essential for application to a unique case at hand.

### Programs with multiple RCTs demonstrating effectiveness

In 2023, the USA federal office for Home Visiting Evidence of Effectiveness (HomVEE) evaluated two attachment‐based programs to have shown sufficient evidence to be eligible for federal funding, ABC and VIPP‐SD (see https://homvee.acf.hhs.gov/ consulted on April 16, 2025).

#### Attachment and Biobehavioral Catch‐up (ABC)

ABC is a 10‐session, in‐home intervention for caregivers with children aged 0–4 years. ABC focuses on promoting caregivers' nurturing care when their children are distressed (safe haven), following their child's lead (secure base), and reducing the caregiver frightening behavior. Although some video‐feedback is used, the key element of ABC is using ‘in the moment’ comments from the parent coach to the caregiver about observed caregiver behaviors. Each time the caregiver follows their child's lead (or not) or shows nurturing behavior when their child is distressed (or fails to), the parent coach comments. In the moment, comments describe the child's behavior and caregiver's response, link the behavior to the intervention target, and link to a potential outcome of the sequence. Parent coaches are expected to make comments in at least 50% of the opportunities during a home visit, with a minimum of one comment per minute. The frequency and quality of in‐the‐moment comments were found to predict the caregiver's increase in sensitivity (Caron, Bernard, & Dozier, 2018).

Results from multiple RCTs demonstrate that parents who received ABC were more sensitive in observed interactions with their children than parents who received an active control condition (e.g., Bick & Dozier, 2013; Garnett, Bernard, Hoye, Zajac, & Dozier, 2020). Children whose parents received ABC formed secure attachments more often and disorganized attachments less often than children of parents in the active control condition (Bernard et al., 2012); they reported more trusting relationships with their parents at age 9 (Zajac, Raby, & Dozier, 2020) and 14 (Miller et al., 2024), with neuroimaging results mirroring these effects (Valadez, Tottenham, Tabachnick, & Dozier, 2020). Children in the ABC group also showed better observed inhibitory control (Lind, Bernard, Yarger, & Dozier, 2020) and executive functioning (Lind, Lee Raby, Caron, Roben, & Dozier, 2017), and more normative cortisol production than children in the control group (e.g., Bernard, Hostinar, & Dozier, 2015; Garnett et al., 2020).

Although effects are often much smaller when moving a program from a university setting to the community, ABC's effect sizes for pre‐ to post‐intervention changes in parental sensitivity are large, with effects similar to those seen through RCTs (e.g., Roben, Dozier, Caron, & Bernard, 2017). To date, no meta‐analysis has combined results from the ABC RCTs. The three federal American clearinghouses (HomVEE, Family First Prevention Services Act [FFPSA], California Evidence‐Based Clearinghouse [CEBC]) have approved and endorsed ABC.

### Video‐based Intervention to promote Positive Parenting and Sensitive Discipline (VIPP‐SD)

VIPP‐SD is meant for families with children up to 7 years (Juffer, Bakermans‐Kranenburg, & Van IJzendoorn, 2017; Van IJzendoorn, Schuengel, Wang, & Bakermans‐Kranenburg, 2023). VIPP‐SD draws on attachment and social learning theories, in particular coercion theory, which explains how ineffective caregiver discipline strategies result in increasingly difficult and challenging child behavior. Instead of rewarding negative child reactions by giving in when confronted with challenging behavior, caregivers are encouraged to consistently and gently reinforce positive behaviors and set rules and limits.

VIPP‐SD consists of six sessions, four sessions with specific themes each, followed by two booster sessions. During each session, the videotaped child–caregiver interaction of the previous session is reviewed with the caregiver, as material to discuss and reflect on the themes of the intervention. In the first session, the difference between child attachment signals and exploration signals, or between secure base and safe haven, is explained. The discipline part focuses on giving explanations for *don'ts* and *do*s and distractions to help children comply. In the second session, caregivers are invited to take the child*'*s perspective using ‘speaking for the child’, and gentle discipline is promoted by introducing positive reinforcement and ignoring negative attention seeking. In the third session, a chain of sensitive interaction is highlighted in the video recordings, comprising a child signal, followed by a caregiver's sensitive response, and the child's reaction to that response. The discipline theme introduces a sensitive interaction pause as a sometimes‐necessary break to de‐escalate conflicts, followed by interactional repair. The fourth session focuses on sharing emotions, affect attunement, and empathy for the child while maintaining clear, consistent limits. In the two booster sessions, the themes are repeated. By focusing on positive moments, caregivers expand their strengths and confidence to try alternative caregiving behaviors when confronted with challenging child behaviors.

VIPP‐SD has demonstrated meta‐analytical effectiveness in promoting positive caregiving and secure child‐caregiver attachment based on 25 RCTs (Van IJzendoorn et al., 2023). The combined effect for sensitive caregiving and discipline, as well as for child attachment security, was moderate but robust, and larger effects on sensitivity led to more attachment security (Van IJzendoorn et al., 2023). Notably, the VIPP‐SD program was effective in enhancing parental sensitive responsiveness in a rural low‐SES Colombian sample (Barone, Carone, Salazar‐Jimenez, & Ortíz Muñoz, 2020). Combined with autism‐specific components, VIPP‐SD also led infants showing early signs of ASD to develop reduced ASD symptom severity and lowered odds of an ASD diagnosis at age 3 years (iBASIS‐VIPP; Whitehouse et al., 2021). The authors showed that the program saved US $3,607 per child as modeled to age 12 years (Segal et al., 2023). Further, when evaluated in a pragmatic RCT and delivered by frontline staff in a routine health service context (i.e., NHS), VIPP‐SD lowered the prevalence of behavioral problems in young children (O'Farrelly et al., 2021). In an RCT, twin children showed less conduct problems and lower hair cortisol concentrations after the VIPP‐SD intervention (Runze, Pappa, Van IJzendoorn, & Bakermans‐Kranenburg, 2022). In 2023, the federal USA clearinghouse (HomVEE) approved and endorsed VIPP‐SD, as did the Netherlands Youth Institute since 2008.

### Promising programs with a smaller evidence‐base

#### Attachment Video‐feedback Intervention (AVI)

AVI (Moss et al., 2011) aims to enhance caregiver sensitivity and child‐caregiver interaction quality to promote child development. Consisting of 8‐weekly 60‐ to 90‐min home visits, it has been evaluated with caregivers and children 0–7 years, with a 7‐ to 12‐year‐old adaptation under investigation. While following a standardized protocol, AVI can be tailored to each family's relational challenges and the organizations' clinical setting. During sessions, caregivers receive immediate video feedback after filmed interactions, with practitioners reinforcing sensitive behaviors and guiding recognition of the child's needs and communications.

Eight RCTs across Canada, Spain, and France support AVI's effectiveness for high‐risk families, revealing greater improvements in parental sensitivity, interaction quality, reflective functioning, and attachment security for AVI participants versus controls (Baudry & Tarabulsy, 2013; Cyr et al., 2022; Dubois‐Comtois et al., 2017; Eguren, Cyr, Dubois‐Comtois, & Muela, 2023; Gómez, & Hosey, 2025; Miljkovitch et al., 2023; Moss et al., 2011). AVI participants showed reduced disengaged parenting, child attachment disorganization, and child behavioral problems. AVI is effective for children in foster care (Dubois‐Comtois, Cyr, Moss, St‐André, & Carignan, 2011), who are internationally adopted (Cyr & Dubois‐Comtois, 2017), and those with autism (Fortin, 2024). Lastly, AVI is a promising tool for informing placement decisions based on assessment of caregiving capacity in cases of child maltreatment (Cyr et al., 2022).

#### Child–Parent Psychotherapy (CPP)

CPP (Lieberman et al., 2005; i.e., Infant‐, Toddler‐, Preschooler‐Parent Psychotherapy, depending on child age) is for children 0–5 years, who have experienced maltreatment, neglect, domestic violence, or other familial hardships. Rooted in the psychodynamic tradition of parent–infant psychotherapy (Fraiberg, 1980), interveners work concurrently with caregivers *and* child, aiming to support mutual affect regulation, and to promote the caregiver's capacity for responding sensitively to the child's difficult feelings and behaviors. CPP integrates attachment theory and psychodynamic theory while incorporating elements from social learning theory, trauma theory, and cognitive behavioral therapy techniques. Intervention length varies based on needs, typically ranging from 22 to 45 weekly one‐hour sessions (e.g., Ghosh Ippen, Harris, Van Horn, & Lieberman, 2011; Toth, Rogosch, Manly, & Cicchetti, 2006).

Five RCTs have evaluated CPP; three assessed attachment outcomes, with two reporting significant increases in attachment security and reductions in attachment disorganization among children receiving CPP (Cicchetti, Rogosch, & Toth, 2006; Toth et al., 2006). Positive CPP effects have also been reported regarding children's posttraumatic stress symptoms (Lieberman et al., 2005), cognitive development (Cicchetti, Rogosch, & Toth, 2000), mental representations of self and caregivers (Toth et al., 2006), and behavior problems (Lieberman et al., 2005; Lieberman, Ghosh Ippen, & Van Horn, 2006). Regarding caregivers, the extant research found improvements in caregiver distress symptoms (Lieberman et al., 2005, 2006); caregiver responsiveness and ability to initiate child interactions (Lieberman, Weston, & Pawl, 1991); and reductions in stress (Lieberman et al., 2006; Toth, Sturge‐Apple, Rogosch, & Cicchetti, 2015).

#### Circle Of Security (COS)

The two most well‐known COS interventions are COS‐Intensive (COS‐I; Powell et al., 2013) and COS‐Parenting (COS‐P; Cooper et al., 2009). Both interventions work with caregivers to improve understanding of their children's needs, enhance their ability to observe and interpret behaviors, recognize their own reactions to their children's behavior, regulate their emotional responses, and develop sensitive parenting practices while learning to repair disruptions in responsiveness.

COS‐I is a 20‐week group‐based intervention. It includes an individualized and comprehensive assessment, with the intervention using video feedback of recorded interactions between the caregiver and their child to enhance the caregiver's understanding of their child's attachment and exploration cues. Meta‐analytic data suggested that COS‐I increases child attachment security, quality of caregiver, and caregiver self‐efficacy, while decreasing caregiver depression; however, only two studies used a randomized control design (Yaholkoski, Hurl, & Theule, 2016).

COS‐P is an 8–10 week, lower‐intensity group‐based psychoeducational intervention. In COS‐P, caregivers reflect on pre‐selected videos of other child‐caregiver interactions rather than their own. In a waitlist control RCT, Cassidy et al. (2017) found COS‐P reduced caregivers' unsupportive responses to their children's distress. However, COS‐P did not influence child attachment or problem behaviors. More recently, a randomized waitlist control study found that COS‐P enhances caregiver reflective functioning, but did ‘not demonstrate significant growth in caregiver behaviors known to promote secure attachment’ (Dexter & Wong, 2024). Null findings were also reported in three recent RCTs (Risholm Mothander, Furmark, & Neander, 2018; Rosan et al., 2025; Zimmer‐Gembeck et al., 2022).

#### Group Attachment‐Based Intervention (GABI)

GABI is a trauma‐informed, multi‐family intervention intended to prevent maltreatment for families with children up to 3 years (Steele et al., 2019). In this group‐based intervention, families meet three times a week for 2 hours over 26 weeks, with each session including dyadic, caregiver‐only, and child‐only elements. GABI provides 24/7 clinical team text access and has a flexible schedule, accommodating families' unpredictable schedules. Delivered in a clinical setting, GABI aims to allow the intervention and the setting itself to serve as a secure base. The goal of GABI is to promote attachment security and prevent disorganized attachment through enhancing the child‐caregiver relationship, treating the child, caregiver, and relationship concurrently. In an RCT, GABI showed significant improvements in the child‐caregiver relationship (Steele et al., 2019). Compared to a treatment‐as‐usual control group, GABI enhanced maternal sensitivity and dyadic reciprocity and was linked to reductions in maternal hostility and risk of later child maltreatment. GABI is currently being delivered in every borough in New York City, with local government funding.

#### Minding The Baby (MTB)

MTB is a mentalization‐based treatment conducted in the home, designed to enhance developmental outcomes for families with their first child (Slade et al., 2023). MTB commences in pregnancy and ends at the child's second birthday, running weekly. In two RCTs, MTB participation was associated with greater caregiver reflective functioning and secure attachment, and lower rates of disorganized attachment (Sadler et al., 2013; Slade et al., 2020). Follow‐up research revealed more supportive parenting, less impaired mentalizing in MTB mothers, and reduced child behavioral problems (Londoño Tobón et al., 2022). Other follow‐up studies reported lower rates of obesity in MTB toddlers (Ordway et al., 2018) and lower levels of C‐reactive protein in MTB children at school age (Londoño Tobón et al., 2023). An RCT found mixed results (Longhi et al., 2019): while MTB showed no effect on parental sensitivity, attachment security, or cognitive development, it reduced behavior problems in two‐year‐olds. More broadly, recent meta‐analytic evidence indicates uncertainty about whether MTB and similar mentalization‐based interventions effectively influence parent–child interaction (Sleed, Li, Vainieri, & Midgley, 2023). Further research is needed to resolve these questions.

### Core components of attachment‐informed intervention programs

No attachment‐informed intervention can be called *the* attachment intervention or attachment therapy par excellence. Ultimately, attachment‐informed interventions work by helping caregivers recognize and modify their interactive patterns to better read, respond to, and ultimately meet their children's attachment needs. Important components of attachment‐based interventions include exploring caregivers' perspectives; supporting caregiver mind‐mindedness and perspective‐taking; providing in‐the‐moment feedback; stimulating caregivers' reflection on video‐recorded interactions with their own child; and showing ways to gently but consistently set boundaries in case of challenging child behavior.

From a strict translational perspective, the practitioner is invited to follow the time‐tested protocol of an evidence‐based intervention module in addition to creative care‐as‐usual based on their vast expertise‐by‐experience. From an expansive translational perspective, practitioners may use elements of evidence‐based interventions to provide support to reduce frightening or hostile caregiving behaviors, promote secure base and safe haven provision, help caregivers understand the importance of the attachment network, and address the caregiver's attachment history that may influence their caregiving.

For all attachment‐informed intervention programs, ethical grounding might be found in cross‐cultural studies documenting the desirability of sensitive parenting and secure attachment for parents in a large variety of cultures. However, from a strict translational perspective, more cultural specificity of desirability of goals and means is needed. The Learning Community approach might lead to a shared ethical basis and, through close cooperation between practitioners and researchers, stimulate sustainable implementation of attachment‐informed programs at scale (Berlin et al., 2016). Cost‐effectiveness has also been rarely studied in attachment‐informed trials and badly needs input from clinical researchers, practitioners, and parents (Van IJzendoorn & Bakermans‐Kranenburg, 2024). A firmer bridge across the attachment research‐practice divide is required to address these outstanding questions, and although the type of cooperation is debated (Bakermans‐Kranenburg & Van IJzendoorn, 2024; Madigan et al., 2024), an ongoing dialog between researchers and practitioners is crucial.

## Conclusion

In this practitioner review, we summarized generally endorsed key findings from attachment theory and research and addressed misconceptions to provide guidance for policymakers and professionals working with children and their caregivers. The state‐of‐the‐art review should be considered in the stage of discovering common ground between experts from various disciplines and professions without an unambiguously replicable methodology of literature search, risk of bias assessment, and formal analysis of the evidence. This review is therefore limited in that it presents hypotheses and conjectures that should be followed up by systematic attempts to test and refute (or provisionally accept) those fallible conjectures (Van IJzendoorn & Bakermans‐Kranenburg, 2024). It is a call for closer cooperation between experts from both research and practice.

We provided an overview of common attachment‐related measures and discussed ‘strict’ and ‘expansive’ translational perspectives on their usefulness for practitioners. We then reviewed a number of evidence‐based interventions and distinguished between strict and expansive translational perspectives on their application. The strict translational perspective emphasizes the evidence‐based limitations of what attachment measures and interventions can offer to practitioners. Yet much of practitioners' work necessarily relies on a vast reserve of expertise‐by‐experience with the treatment of troubled families, and their creativity to find the best courses of action right now. According to the expansive translational perspective, clinical practice may benefit from a more flexible use of scientific findings, where practitioners may be inspired or sharpen their insights through knowledge of attachment measures or interventions. In this view, following training in attachment or parenting measures may benefit practitioners' capacity for observation and for formulating clinical hypotheses. For their clinical practice, using elements of evidence‐based interventions in an eclectic way may be helpful even if demonstrated effectiveness has become moot.

For children, the attachment principles address their core needs for safe relationships where they can typically express attachment signals, explore their environment, and receive consistent (ideally also sensitive) responses from caregivers. For caregivers, providing such care is not always easy or evident, and some parents, or parents in some conditions, need support to provide ‘good‐enough’ parenting (Forslund et al., 2022). Although a strong evidence base is lacking, the same ‘secure base skills’ that are applicable to caregivers might also work for the therapeutic relationship itself (Bowlby, 1988; Byng‐Hall, 2008) as they include promoting client autonomy and agency. Independence is something that may grow out of secure relationships within therapy as well as within families, not as its opposite. The ideals of the ‘good enough’ practitioner are leading as they involve acceptance of imperfection while striving for effectiveness and improvement (Holmes & Slade, 2017). The same acceptance of imperfection and striving for improvement may characterize attachment researchers. The common goal of all stakeholders is to provide vulnerable children with the safe, stable, and shared family‐based care they need.

## Ethical considerations

Not applicable – no data was collected for the present practitioner review.

**Key points**

**What's known?**

- While attachment theory is widely used by practitioners, its translation into community and clinical practice can be challenging. This manifests in attachment misconceptions, misinterpretations, and inconsistency of application.

**What's new?**

- Authored by an international team of 47 attachment researchers and practitioners, ensuring both scientific rigor and clinical relevance, the review outlines attachment propositions based on contemporary replicated research, addresses common misconceptions hindering practical applications, and presents information on measures of attachment and sensitive parenting that can be useful for practitioners. It also reviews evidence‐based and promising attachment interventions, discussing core components of (preventive) support for parents or caregivers and the children in their care.

**What's relevant?**

- ‘Strict’ and ‘expansive’ translational approaches to applying attachment measures and interventions are introduced. The strict perspective emphasizes the evidence‐based limitations of what attachment measures and interventions can offer practitioners, while the expansive approach supports flexible use of scientific findings and measures to enhance practitioners’ observational skills and clinical hypothesis formation.
- Attachment theory's clinical value lies *not* in assigning classifications, but in understanding crucial insights into caregiving and early socioemotional development.
- Children need safe, stable, and shared family‐based care arrangements (‘Triple S care’) enabling attachment signal expression, increasing self‐reliant exploration, and consistent caregiver responses, while parents receive support for ‘good‐enough’ parenting in a network of attachment figures.
- Secure base skills applicable to caregiving may enhance therapeutic relationships by promoting client autonomy. The ‘good enough’ practitioner ideal involves accepting imperfection while striving for effectiveness.

## Supporting information


**Appendix S1.** Suggested practitioner resources.

## Data Availability

Data sharing is not applicable to this article as no datasets were generated or analyzed.
