# Supplementary material for: Practitioner Review: Clinical insights from attachment theory and research for professionals working with young children and their families
Source: J Child Psychol Psychiatry. 2026 Feb 25;67(5):723–39. doi: 10.1111/jcpp.70126 (PMC13102048; doi:10.1111/jcpp.70126)
Supplement: Supplementary file 1 — Appendix S1. Suggested practitioner resources. [file JCPP-67-723-s001.docx]

**Practitioner Review: Clinical Insights from Attachment Theory and Research for Professionals Working with Young Children and their Families**

**Supporting Information**

**Appendix S1. SUGGESTED PRACTITIONER RESOURCES**

| **Books** |
| --- |
| ***Featured:*** Duschinsky, R., Forslund, T., & Granqvist, P. (2023). *The psychology of attachment.* Routledge.  Allen, B. (2023). *The science and clinical practice of attachment theory: A guide from infancy to adulthood.* American Psychological Association.  Cassidy, J., & Shaver, P.R. (Eds.). (2016). *Handbook of attachment: Theory, research, and clinical applications* (3rd ed.). Guilford Press.  Duschinsky, R. (2025). *Developments in attachment research*. Oxford University Press. Free PDF download from the Oxford University Press Site: [https://academic.oup.com/book/60504](https://protect.checkpoint.com/v2/___https://academic.oup.com/book/60504___.YzJ1OnN0b255YnJvb2s6YzpnOjkyMDQwMzJkMWFkOWVmYzg5MjM1ZGY2ODZjZDc0OTFiOjY6OTJiYzo3ODE3ZGZkM2IyOGExM2EyYTNmMmQ3OGI0Zjg0MjdlMjIzYjBhZjY0NDc1ZGI5MGQ0ZTJkZWZkNmVkMjIzZTllOnA6VDpO)  Duschinsky, R. (2020). *Cornerstones of attachment research*. Oxford University Press. Free PDF download: [https://academic.oup.com/book/28760](https://protect.checkpoint.com/v2/___https://academic.oup.com/book/28760___.YzJ1OnN0b255YnJvb2s6YzpnOjkyMDQwMzJkMWFkOWVmYzg5MjM1ZGY2ODZjZDc0OTFiOjY6YzRkODo4Zjc2ZDA1NmQ5NzNlNDlkM2ZjOWI3MjQzNWQyODgzOWE1NDFjNTc3NjM5MDQ4ZmYzZDgzNzI2MTU5NjcxZDVjOnA6VDpO)  Holmes, J., & Slade, A. (2017). *Attachment in therapeutic practice.* Sage.  Mikulincer, M., & Shaver, P. R. (2023). *Attachment theory applied: Fostering personal growth through healthy relationships.* The Guilford Press.  Mucha, L. (2025) *Please find attached: How attachment theory explains our relationships*. Bloomsbury  Sroufe, A., & Sroufe, J. (2025). *The development and organization of meaning: How individual worldviews develop in relationships.* Cambridge University Press.  Steele, H., & Steele, M. (Eds.). (2018). *Handbook of attachment-based interventions*. The Guilford Press.  Osofsky, J. D., Fitzgerald, H. E., Keren, M., & Puura, K. (Eds.). (2024). *WAIMH handbook of infant and early childhood mental health: Biopsychosocial factors: Volume one.* Springer. [https://doi.org/10.1007/978-3-031-48627-2](https://protect.checkpoint.com/v2/___https://doi.org/10.1007/978-3-031-48627-2___.YzJ1OnN0b255YnJvb2s6YzpnOjkyMDQwMzJkMWFkOWVmYzg5MjM1ZGY2ODZjZDc0OTFiOjY6Mjg4NTowNTE1NjVlNzAxOGFmNDk1NWM5N2Y1YmMzODYzNmJjNmZmZjRlYjA2ZDVlNjEyZGNkMWExM2U0NzcxOGZlYmZlOnA6VDpO)  Thompson, R. A., Simpson, J. A., & Berlin, L. J. (2021). *Attachment: The fundamental questions.* New York: Guilford.  Van IJzendoorn, M. H., & Bakermans-Kranenburg, M.J. (2024). *Matters of significance*. London, UK: UCL Press. Free PDF download: [https://uclpress.co.uk/book/matters-of-significance/](https://protect.checkpoint.com/v2/___https://uclpress.co.uk/book/matters-of-significance/___.YzJ1OnN0b255YnJvb2s6YzpnOjkyMDQwMzJkMWFkOWVmYzg5MjM1ZGY2ODZjZDc0OTFiOjY6NTg3Yzo5ODk1MmViZTY3MTEzOTMxODBlMzY5ZDIxYjEwMzIzYTQwZTY3YWI2ZjRlY2EyMzNlY2E0Nzc3N2EzZGU3NDI3OnA6VDpO)  Waters, E., Vaughn, B. E., & Waters, H. S. (2021). *Measuring attachment: Developmental assessment across the lifespan.* Guilford Press.  Zeanah, C. H. (Ed.). (2018). *Handbook of infant mental health.* Guilford Publications. |
| **Peer-reviewed articles** |
| ***Featured:*** Thompson, R. A., Simpson, J. A., & Berlin, L. J. (2022). Taking perspective on attachment theory and research: nine fundamental questions. *Attachment & Human Development*, *24*(5), 543–560. Free PDF download: [https://doi.org/10.1080/14616734.2022.2030132](https://protect.checkpoint.com/v2/___https://doi.org/10.1080/14616734.2022.2030132___.YzJ1OnN0b255YnJvb2s6YzpnOjkyMDQwMzJkMWFkOWVmYzg5MjM1ZGY2ODZjZDc0OTFiOjY6ZGYxYTpiYzlhMDFlODQ5YTRkMzc3YWE2MWJjYWI1ZTU4NmYzMTZkNDg1NzFhMzcwYzNlODUyM2FmZjNjODgwN2I0M2Q4OnA6VDpO)  ***Featured:*** Granqvist, P., Sroufe, L. A., Dozier, M., Hesse, E., Steele, M., Van IJzendoorn, M., Solomon, J., Schuengel, C., Fearon, R.M.P., Bakermans-Kranenburg, M., Steele, H., Cassidy, J., Carlson, E., Madigan, S., Jacobvitz, D., Foster, S., Behrens, K., Rifkin-Graboi, A., Gribneau, N., Spangler, G., … Duschinsky, R. (2017). Disorganized attachment in infancy: a review of the phenomenon and its implications for clinicians and policy-makers. *Attachment & Human Development*, *19*(6), 534–558. Free PDF download: [https://doi.org/10.1080/14616734.2017.1354040](https://protect.checkpoint.com/v2/___https://doi.org/10.1080/14616734.2017.1354040___.YzJ1OnN0b255YnJvb2s6YzpnOjkyMDQwMzJkMWFkOWVmYzg5MjM1ZGY2ODZjZDc0OTFiOjY6MjdkYzo0OWEyY2FjYjY0ZjIzMmIyZDRmYzE3MTQzNzJjYTY1MDE2MzU3OGM1MDk2ODNiMDY4OTEyOWRmMjVhNGZhZGM4OnA6VDpO)  Bakermans-Kranenburg, M.J. & Van IJzendoorn, M.H. (2024). Family matters. Growing up in family-based care makes a world of a difference. Proceedings of the Shevchenko Scientific Society. *Medical Sciences*, *73*(1). Free PDF download: [https://doi.org/10.25040/ntsh2024.01.05](https://protect.checkpoint.com/v2/___https://doi.org/10.25040/ntsh2024.01.05___.YzJ1OnN0b255YnJvb2s6YzpnOjkyMDQwMzJkMWFkOWVmYzg5MjM1ZGY2ODZjZDc0OTFiOjY6YzkyNzo0ODU4ZjZiNjc1MGEyNjBmMDMxODg5N2QwOWY3NDM0MDE4MmQwMGM5NDk3YjRiOGEzOGJlYmJmOTllZGFiMWY0OnA6VDpO)  Forslund, T., Granqvist, P., Van IJzendoorn, M. H., Sagi-Schwartz, A., Glaser, D., Steele, M., Hammarlund, M., Schuengel, C., Bakermans-Kranenburg, M. J., Steele, H., Shaver, P. R., Lux, U., Simmonds, J., Jacobvitz, D., Groh, A. M., Bernard, K., Cyr, C., Hazen, N. L., Foster, S., . . . Duschinsky, R. (2022). Attachment goes to court: child protection and custody issues. *Attachment & Human Development*, *24*(1), 1–52. Free PDF download: [https://doi.org/10.1080/14616734.2020.1840762](https://protect.checkpoint.com/v2/___https://doi.org/10.1080/14616734.2020.1840762___.YzJ1OnN0b255YnJvb2s6YzpnOjkyMDQwMzJkMWFkOWVmYzg5MjM1ZGY2ODZjZDc0OTFiOjY6NDI0YToyMGU3ZjNhYzVjODBmMGQ2NzJkYmM3ZmVhNzlmZjcxOGY5ZmM0OWZjYjk4YWIyMzMwYzZlZDNlZjk3YWRmYWRhOnA6VDpO)  Slade, A., & Holmes, J. (2019). Attachment and psychotherapy. *Current Opinion in Psychology*, *25*, 152–156. [https://doi.org/10.1016/j.copsyc.2018.06.008](https://protect.checkpoint.com/v2/___https://doi.org/10.1016/j.copsyc.2018.06.008___.YzJ1OnN0b255YnJvb2s6YzpnOjkyMDQwMzJkMWFkOWVmYzg5MjM1ZGY2ODZjZDc0OTFiOjY6ZWM0ODozNWVhOWVlZDMxOTQ2M2FiMzc1MGNkMTliZjhmZTRhZWYwMjM4ODgzNTdkOWFjNjBjYWVjMjQ4Y2VkMTA4ZjkyOnA6VDpO)  Van IJzendoorn, M.H., & Bakermans-Kranenburg, M.J. (2021). Replication crisis lost in translation? On translational caution and premature applications of attachment theory. *Attachment & Human Development*, *23*(4), 422–437. Free PDF download: [https://doi.org/10.1080/14616734.2021.1918453](https://protect.checkpoint.com/v2/___https://doi.org/10.1080/14616734.2021.1918453___.YzJ1OnN0b255YnJvb2s6YzpnOjkyMDQwMzJkMWFkOWVmYzg5MjM1ZGY2ODZjZDc0OTFiOjY6NzY1NDo3NGNlYTQyZjc1MTNlOTRhNDE4YThkMTkxODQ0NjAwZjQxZTZkZTZlYzQwZWU0MjU5OThjNDc2NzdhOTkyZTRhOnA6VDpO)  Verhage, M. L., Tharner, A., Duschinsky, R., Bosmans, G., & Fearon, R. M. P. (2023). Editorial Perspective: On the need for clarity about attachment terminology. *Journal of Child Psychology and Psychiatry, and Allied Disciplines*, *64*(5), 839–843. Free PDF download: [https://doi.org/10.1111/jcpp.13675](https://protect.checkpoint.com/v2/___https://doi.org/10.1111/jcpp.13675___.YzJ1OnN0b255YnJvb2s6YzpnOjkyMDQwMzJkMWFkOWVmYzg5MjM1ZGY2ODZjZDc0OTFiOjY6ZjZjYjo3MzE0YTdkY2Q3YzUwYzFhMzkyNDY0NzdiNjVkY2ZhN2ZkYTAzZmJiMzkzY2YwNWQ2YzRkMDllOWFiODE0M2FkOnA6VDpO) |
| **Classic conceptual texts** |
| ***Featured:*** Slade, A. (2004). The Move from Categories to Process: Attachment Phenomena and Clinical Evaluation. *Infant Mental Health Journal*, *25*(4), 269–283. [https://doi.org/10.1002/imhj.20005](https://protect.checkpoint.com/v2/___https://doi.org/10.1002/imhj.20005___.YzJ1OnN0b255YnJvb2s6YzpnOjkyMDQwMzJkMWFkOWVmYzg5MjM1ZGY2ODZjZDc0OTFiOjY6YWQ5YTozYTY1YjkxZjUwMTI0MzFiM2Y4NTllZjI4ZWRjYTA3NGQ5Y2IzMWE4NWVlNGFjMjZkZjJkYTQxZjg2ZGMwYmJkOnA6VDpO)  Brandt, K., Perry, B. D., Seligman, S., & Tronick, E. (Eds.). (2014). *Infant and early childhood mental health: Core concepts and clinical practice.* American Psychiatric Publishing.  Bretherton, I. (1992). The origins of attachment theory: John Bowlby and Mary Ainsworth. *Developmental Psychology*, *28*(5), 759–775. [https://doi.org/10.1037/0012-1649.28.5.759](https://protect.checkpoint.com/v2/___https://doi.org/10.1037/0012-1649.28.5.759___.YzJ1OnN0b255YnJvb2s6YzpnOjkyMDQwMzJkMWFkOWVmYzg5MjM1ZGY2ODZjZDc0OTFiOjY6ZTBjNjoxYmZlYWI4MWU0MzBkZGExZjA2YWZhOTI4Mjk1YTY3NzIyYjYyOTMwYjc3MmZjYjdmMTUzYzAzYzVkYTU1ZTEzOnA6VDpO)  Belsky, J., Bakermans-Kranenburg, M. J., & Van IJzendoorn, M. H. (2007). For Better and For Worse: Differential Susceptibility to Environmental Influences. *Current Directions in Psychological Science*, *16*(6), 300-304. [https://doi.org/10.1111/j.1467-8721.2007.00525.x](https://protect.checkpoint.com/v2/___https://doi.org/10.1111/j.1467-8721.2007.00525.x___.YzJ1OnN0b255YnJvb2s6YzpnOjkyMDQwMzJkMWFkOWVmYzg5MjM1ZGY2ODZjZDc0OTFiOjY6ZmVkYzoxNDBhMWJmNjc2OTBiMWFiOWQ3NGIxMTI1Yjc1NGMzN2NlMGJjMWEyYmVlNjkzYTE2ZDJjNDFmMTRjOGVjOWMyOnA6VDpO)  Carlson, E.A., Sroufe, L.A., & Egeland, B. (2004). The construction of experience: A longitudinal study of representation and behavior. *Child Development,* *75*(1), 66-83.  Crowell, J. A. (2003). Assessment of attachment security in a clinical setting: Observations of parents and children. *Journal of Developmental and Behavioral Pediatrics*, *24*(3), 199–204. [https://doi.org/10.1097/00004703-200306000-00012](https://protect.checkpoint.com/v2/___https://doi.org/10.1097/00004703-200306000-00012___.YzJ1OnN0b255YnJvb2s6YzpnOjkyMDQwMzJkMWFkOWVmYzg5MjM1ZGY2ODZjZDc0OTFiOjY6MjM3Yzo0MDk3NjMxZWU1M2Q4ZDY2NTI5NDg3YTEwYmQ3OTA2NjU1NTVhOGRkYmI1ZWI1MWRjMDQyOWU1ODM5NWE5NTg4OnA6VDpO)  Oppenheim, D., & Goldsmith, D. F. (Eds.). (2011). *Attachment theory in clinical work with children: Bridging the gap between research and practice.* Guilford press.  Rutter M. (1995). Clinical implications of attachment concepts: retrospect and prospect. *Journal of Child Psychology and Psychiatry, and Allied Disciplines*, *36*(4), 549–571. [https://doi.org/10.1111/j.1469-7610.1995.tb02314.x](https://protect.checkpoint.com/v2/___https://doi.org/10.1111/j.1469-7610.1995.tb02314.x___.YzJ1OnN0b255YnJvb2s6YzpnOjkyMDQwMzJkMWFkOWVmYzg5MjM1ZGY2ODZjZDc0OTFiOjY6OWZmMTpiYjljZTM0Y2FkMzFjYTM2Y2U5N2ZiNjliNmQ2M2JjODY0ZjVkZjI4YjBjYWM3NzQ0OTkwNWExODQ0YjlkNmJlOnA6VDpO)  Sroufe, L. A., Egeland, B., Carlson, E. A., & Collins, W. A. (2005). *The development of the person: The Minnesota study of risk and adaptation from birth to adulthood.* Guilford Publications.  Zeanah, C. H., Berlin, L. J., & Boris, N. W. (2011). Practitioner review: Clinical applications of attachment theory and research for infants and young children. *Journal of Child Psychology and Psychiatry*, *52*(8), 819–833. [https://doi.org/10.1111/j.1469-7610.2011.02399.x](https://protect.checkpoint.com/v2/___https://doi.org/10.1111/j.1469-7610.2011.02399.x___.YzJ1OnN0b255YnJvb2s6YzpnOjkyMDQwMzJkMWFkOWVmYzg5MjM1ZGY2ODZjZDc0OTFiOjY6ZjI3ZjpmNzJlYjMyMTIwMTc4ZTRmNjEyM2I5NGEzMzc0NTQ4MzViMWQ0YzdkNzc1ZTBmM2M1YTA1ODI0YzE1YmM3Mjk1OnA6VDpO) |
| **Online material written by attachment researchers and practitioners**  *We report these due to the vast amount of misinformation on attachment theory online.* |
| ***Featured:*** Society for Emotion and Attachment Studies. (2021). *Explanations of attachment theoretical concepts.* [https://seasinternational.org/explanations-of-attachment-theoretical-concepts](https://protect.checkpoint.com/v2/___https://seasinternational.org/explanations-of-attachment-theoretical-concepts___.YzJ1OnN0b255YnJvb2s6YzpnOjkyMDQwMzJkMWFkOWVmYzg5MjM1ZGY2ODZjZDc0OTFiOjY6NzRiYzo5YzQwNGE2ODA4ZTc4ZWQ2NjIyNTE4NTczMDFjZTc2M2ViYWIxYjEzZWQwNDk3Y2Y3ODZjZThmZjA2OGU4YmFjOnA6VDpO)  Ainsworth, M. (1969). Ainsworth Maternal Sensitivity Scales. [http://www.psychology.sunysb.edu/attachment/measures/content/ainsworth_scales.html](https://protect.checkpoint.com/v2/___http://www.psychology.sunysb.edu/attachment/measures/content/ainsworth_scales.html___.YzJ1OnN0b255YnJvb2s6YzpnOjkyMDQwMzJkMWFkOWVmYzg5MjM1ZGY2ODZjZDc0OTFiOjY6YTYzMTo1ZDQ4NDM1NGQ3YjMwOTVlMjczNjg4OTlkNDc0ZDgxYjg2YjEyYjY5N2U4NTU4OWFiNmQ3MWE0YmZiZTJjZWY2OnA6VDpO)  Dagan, O., & Schuengel, C. (2023). Secure attachment to both parents − not just mothers − boosts children’s healthy development. *The Conversation.* [https://theconversation.com/secure-attachment-to-both-parents-not-just-mothers-boosts-childrens-healthy-development-213108](https://protect.checkpoint.com/v2/___https://theconversation.com/secure-attachment-to-both-parents-not-just-mothers-boosts-childrens-healthy-development-213108___.YzJ1OnN0b255YnJvb2s6YzpnOjkyMDQwMzJkMWFkOWVmYzg5MjM1ZGY2ODZjZDc0OTFiOjY6ZWQ0ZTpkNDIyZmFlMmMzZWM3ZDVhNDc5ODUzMTcxODM3MzAxNGExYmY0ZjMyYjQzODRmOTUwZGIxOGJiNDlmYTViYjNmOnA6VDpO)  Dagan, O., & Schuengel, C. (2024). Difficult children are only slightly more likely to have insecure attachments with parents. *The Conversation.* [https://theconversation.com/difficult-children-are-only-slightly-more-likely-to-have-insecure-attachments-with-parents-236106](https://protect.checkpoint.com/v2/___https://theconversation.com/difficult-children-are-only-slightly-more-likely-to-have-insecure-attachments-with-parents-236106___.YzJ1OnN0b255YnJvb2s6YzpnOjkyMDQwMzJkMWFkOWVmYzg5MjM1ZGY2ODZjZDc0OTFiOjY6MTg3YToyNmVmZDllOTY4NGEyM2IzN2I4MTkxNjFlODhhY2Y3NzJlZTNlMWM4YzAyZmI0N2ZiYTRmYjMxYTE4ZDk0ZGM5OnA6VDpO)  Duschinsky, R. (2021). Demystifying attachment. *The Psychologist*. [https://www.bps.org.uk/psychologist/demystifying-attachment](https://protect.checkpoint.com/v2/___https://www.bps.org.uk/psychologist/demystifying-attachment___.YzJ1OnN0b255YnJvb2s6YzpnOjkyMDQwMzJkMWFkOWVmYzg5MjM1ZGY2ODZjZDc0OTFiOjY6MDVlNzo0MGYyYWE0NWVjZTYzODc0NzlkMzQ0ODdlY2Y2ZTczYWRmNDM2MmQ2OWFmYTAwNzMzNWQxNjExMjY5ZjRlMjM1OnA6VDpO)  Hammarlund, M., Granqvist, P., & Forslund, T.. (2023). Attachment theory: How an obsession with its classifications can harm children. *The Conversation*. [https://theconversation.com/attachment-theory-how-an-obsession-with-its-classifications-can-harm-children-202903](https://protect.checkpoint.com/v2/___https://theconversation.com/attachment-theory-how-an-obsession-with-its-classifications-can-harm-children-202903___.YzJ1OnN0b255YnJvb2s6YzpnOjkyMDQwMzJkMWFkOWVmYzg5MjM1ZGY2ODZjZDc0OTFiOjY6NzZkODo1M2E2N2U3YTk2NzA0ZjA0OGY0YzFlZGY5ZTBjNmZhOWM2ZDI0MjgyMWVmMDVlZWJiN2I1YjcwYTdjYmEzMGM4OnA6VDpO)  Madigan, S. (2023). How children's secure attachment sets the stage for positive well-being. *The Conversation*. [https://theconversation.com/how-childrens-secure-attachment-sets-the-stage-for-positive-well-being-213423](https://protect.checkpoint.com/v2/___https://theconversation.com/how-childrens-secure-attachment-sets-the-stage-for-positive-well-being-213423___.YzJ1OnN0b255YnJvb2s6YzpnOjkyMDQwMzJkMWFkOWVmYzg5MjM1ZGY2ODZjZDc0OTFiOjY6MzNlYToxOTU1MzA4YzFiN2VhMGE4MDhjMWM5YWRlZWRkOTgyMmI2MWI5Yjc3ZTVkNDZjODUwY2UwNjA5ZmZjNzU1OTJlOnA6VDpO)  Madigan, S. (2024). The key to raising secure children: Why parental sensitivity matters for fathers and mothers. *The Conversation*. <https://theconversation.com/the-key-to-raising-secure-children-why-parental-sensitivity-matters-for-fathers-and-mothers-230081> |
| **Freely available online videos** |
| ***Featured:*** Duschinsky, R., Sroufe, L. A., Dozier, M., Hesse, E., Granqvist, P., Steele, M. (2017). Infant disorganized attachment: The key questions [Video]. *YouTube*. [https://www.youtube.com/watch?v=UY7hhVvKGoo](https://protect.checkpoint.com/v2/___https://www.youtube.com/watch?v=UY7hhVvKGoo___.YzJ1OnN0b255YnJvb2s6YzpnOjkyMDQwMzJkMWFkOWVmYzg5MjM1ZGY2ODZjZDc0OTFiOjY6NWNmNTo3ZTIzYzM2Y2EzYjdiMmZhMzk5N2FiODM5ZmZhODE2NTVmOWFiMGI1N2RjOTg5ZTFjZTM1MTc0Y2ZiYTUyZGMzOnA6VDpO)  Granqvist, P. (2017). Attachment and parents with intellectual disabilities [Video]. *YouTube*. [https://www.youtube.com/watch?v=-zkP8PyvECw](https://protect.checkpoint.com/v2/___https://www.youtube.com/watch?v=-zkP8PyvECw___.YzJ1OnN0b255YnJvb2s6YzpnOjkyMDQwMzJkMWFkOWVmYzg5MjM1ZGY2ODZjZDc0OTFiOjY6MWQ1Mjo5YmEyYTg5OTdmZmQ5YjRlMThjNWYxY2I4MWU1MGUzMzQzYzVkNTk0NThmOGY1YTM0NDZiM2UyODViMGQwNjAzOnA6VDpO)  Holmes, J. (2020). John Bowlby, attachment theory, and psychotherapy [Video]. *YouTube*. [https://www.youtube.com/watch?v=-Se9HZoCUOQ](https://protect.checkpoint.com/v2/___https://www.youtube.com/watch?v=-Se9HZoCUOQ___.YzJ1OnN0b255YnJvb2s6YzpnOjkyMDQwMzJkMWFkOWVmYzg5MjM1ZGY2ODZjZDc0OTFiOjY6Njc3ZDphY2IwYTFkNDExYjk2MWM0ZGNlOWJlMzdmMGZiNGUzZDIxZmZlOGFmNDJmNDk5ZTIzZmI0M2Q2ODI5ZTAxNjQ1OnA6VDpO)  Jacobvitz, D. (2017). Intergenerational transmission of attachment [Video]. *YouTube*. [https://www.youtube.com/watch?v=KKoptLJbvmU](https://protect.checkpoint.com/v2/___https://www.youtube.com/watch?v=KKoptLJbvmU___.YzJ1OnN0b255YnJvb2s6YzpnOjkyMDQwMzJkMWFkOWVmYzg5MjM1ZGY2ODZjZDc0OTFiOjY6ZDdkYjo0NjBkZGRkMDAyNTM0YWJjOTlkMzM0YTkyYzdjYWJmZGJjZjYwNWJkY2M3NDAwM2ZmODk3YTBjNDYyNzVkZGFkOnA6VDpO)  Sroufe, L. A. (2018). How we come to define ourselves, attachment research over decades [Video]. *YouTube*. [https://www.youtube.com/watch?v=bgqBelXr1bE](https://protect.checkpoint.com/v2/___https://www.youtube.com/watch?v=bgqBelXr1bE___.YzJ1OnN0b255YnJvb2s6YzpnOjkyMDQwMzJkMWFkOWVmYzg5MjM1ZGY2ODZjZDc0OTFiOjY6Zjg5NjpmMzFjNTM1NDRhNmM5ZWUyM2NhNWU1MmIxYmE5OTAyNDUzMWNjY2IyNGJiOGE3NjJmNzU1ZTQwZTcxNWQ5NGIxOnA6VDpO)  Steele, H., & Foster, S. (2017). Attachment and reflective functioning [Video]. *YouTube*. [https://www.youtube.com/watch?v=PYZBUiqC1g0](https://protect.checkpoint.com/v2/___https://www.youtube.com/watch?v=PYZBUiqC1g0___.YzJ1OnN0b255YnJvb2s6YzpnOjkyMDQwMzJkMWFkOWVmYzg5MjM1ZGY2ODZjZDc0OTFiOjY6Y2I3NDplZjRiZjhhMWIwOWY2ZGNjOGQ2ZDBjN2IxZGJjNmUzOTA1NGEzMjdjODkyNzI1OWFiMTljMmJjOTIwYzFiYmJlOnA6VDpO)  Van IJzendoorn, M. (2018). The idea of attachment (Parts 1-2) [Video]. *YouTube*. [https://www.youtube.com/watch?v=jdmyvrZOdX0](https://protect.checkpoint.com/v2/___https://www.youtube.com/watch?v=jdmyvrZOdX0___.YzJ1OnN0b255YnJvb2s6YzpnOjkyMDQwMzJkMWFkOWVmYzg5MjM1ZGY2ODZjZDc0OTFiOjY6YTVmNjozZWJjYzlmZjFlYjkyMzM0YTZkM2Q5MzdjNTgzMWY1ZjZjYjAzOTlkMjAyNzFiZjkzMTEyY2RkMjg5OTM0OTRiOnA6VDpO)  [https://www.youtube.com/watch?v=JZABlKQajjo](https://protect.checkpoint.com/v2/___https://www.youtube.com/watch?v=JZABlKQajjo___.YzJ1OnN0b255YnJvb2s6YzpnOjkyMDQwMzJkMWFkOWVmYzg5MjM1ZGY2ODZjZDc0OTFiOjY6YmJhYTo2ZDJjNmY5MWJkMGUyZGEyZGQyOGM4Yzg5Mjk3YzBmYmNkZDk1Y2E0MzUyM2U3MDQ2NTgwNjM2ZDQyMGNlNTlhOnA6VDpO) |
| **Continued Training and Education Recommendations**  *It can be difficult to bridge the gap between theoretical knowledge about attachment concepts and the practical ability to recognize these concepts in observed behavior. To help enhance both attachment knowledge and observational skills, we suggest the following training and education resources.* |
| **Ainsworth’s Strange Situation Procedure (SSP; secure, avoidant, resistant)**  **About:** In this training participants will learn: 1) Procedures for conducting the Ainsworth Strange Situation assessment; 2) The Ainsworth approach to coding infant interactive behavior; 3) The Ainsworth ABC classification system; 4) Multiple examples of the various classifications of attachment; 5) Actual experience in coding and classifying cases; and 6) An introduction to “disorganized” attachment. This training includes 35 training cases and additional practice cases. It is comprehensive and carefully explains how to code each training case.  **Cost:** $600  **Time:** 30 hours  **Modality:** Asynchronous online  **Website:** [www.safersociety.org/continuing-education/fundamentals-of-infant-attachment-assessment-the-ainsworth-method/](https://protect.checkpoint.com/v2/___https://safersociety.org/continuing-education/fundamentals-of-infant-attachment-assessment-the-ainsworth-method/___.YzJ1OnN0b255YnJvb2s6YzpnOjkyMDQwMzJkMWFkOWVmYzg5MjM1ZGY2ODZjZDc0OTFiOjY6NmEzMDo3MzAwN2RiMjE5MTM5NTI2OTU3MTVkN2Y0ZGQ1MGRiNDdjMzczZTZlN2Y2ZWI0YjRlYTk5MjdjYjVjOGVkZDQ0OnA6VDpO)  **Presenters:** Profs. Alan Sroufe & Robert Weigand   - Alan Sroufe, Professor Emeritus, University of Minnesota Institute of Child Development is an internationally recognized expert on early attachment relationships, emotional development, and developmental psychopathology, he has published 150 articles and seven books. - Robert Weigand, Emeritus Principal Lecturer of Family and Human Development at Arizona State University, taught early childhood intervention, infant mental health, and child development courses, and was co-director of the Master of Advanced Study in Infant-Family Practice degree program.   **Organization:** Safer Society Foundation  **Contact:** [info@safersociety.org](mailto:info@safersociety.org)  **Attachment Disorganization Training**  Ainsworth’s Strange Situation Procedure (SSP; attachment disorganization)  **About**: In this training participants will: 1) review the Ainsworth ABC classification system; 2) learn the Main and Solomon approach to coding infant attachment disorganization with video-recorded examples; and 3) practice coding and classifying infant attachment categories (ABCD).  **Time**: 30 hours  **Modality**: Synchronous and asynchronous online  **Presenters**: Elizabeth A Carlson & Robert Weigand  ● Elizabeth Carlson, PhD, LP, Senior Research Associate, University of Minnesota Institute of Child Development and Licensed Psychologist, is an internationally recognized expert on early attachment relationships and developmental psychopathology.  ● Robert Weigand, Emeritus Principal Lecturer of Family and Human Development at Arizona State University, is former co-director of the Master of Advanced Study in Infant-Family Practice.  **Contact**: Elizabeth A Carlson ([carls032@umn.edu)](mailto:carls032@umn.edu)  **Adult Attachment Interview (AAI) Institute**  **About**: This training focuses on learning (1) the 9-point dimensional scores for 'probable childhood experiences' with mother and with father (or other primary caregiver); (2) 'current state of mind regarding attachment' with a central focus on coherence; and (3) to make categorical judgments that classify each AAI as either Autonomous-Secure or Insecure (Dismissing or Preoccupied). An additional vital consideration is whether an interview shows signs of unresolved loss or unresolved abuse/trauma. An Unresolved status may be assigned to any interview whether it is Secure or Insecure.  **Cost for the two-week training, and follow-up reliability test:** $3000  **Time**: Two-weeks full-time (60 hours)  **Modality**: Synchronous online (via Zoom), or in-person  **Website**: [www.mainattachment.org](https://protect.checkpoint.com/v2/___http://www.mainattachment.org___.YzJ1OnN0b255YnJvb2s6YzpnOjkyMDQwMzJkMWFkOWVmYzg5MjM1ZGY2ODZjZDc0OTFiOjY6MTE5MTplMjdmZjJmYjNhY2NlNjY5MWMzODU3OWQ4ZWFkNGU4Zjc4YzY2NzRhNTM5ZTVlZWNjZjI5YzIzMWUzZWFmMjAxOnA6VDpO)  **Who**: Taught by members of the Consortium of AAI Trainers, who spent two weeks with Mary Main and Erik Hesse learning how to teach their AAI scoring system, the extensively validated approach to AAIs.  **Contact**: Prof. Howard Steele ([SteeleH@newschool.edu](mailto:SteeleH@newschool.edu))  **The Atypical Maternal Behaviour Instrument for Assessment and Classification.**  This training provides comprehensive instruction in the AMBIANCE (Atypical Maternal Behavior Instrument for Assessment and Classification, and AMBIANCE– brief version), a validated observational tool designed to assess disrupted parental behaviors associated with infant disorganized attachment. Participants will learn:  1. Theoretical foundations and development of the AMBIANCE measure  2. The most salient behavioral indicators of disrupted caregiving  3. How to apply the five dimensions of disrupted parenting (affective communication errors, role confusion, negative-intrusive behavior, disorientation, and withdrawal)  4. Coding and scoring of specific caregiver behaviors  5. Conducting and passing a reliability test to ensure coding fidelity    **Full AMBIANCE Measure**  **Cost:** $1500 **Time:** 30 hours  **Modality:** Asynchronous online    **AMBIANCE-brief measure**  **Cost:** $600 **Time:** 30 hours  **Modality:** Asynchronous online  **Website:** [https://www.madiganlab.com/training](https://protect.checkpoint.com/v2/___https://www.madiganlab.com/training___.YzJ1OnN0b255YnJvb2s6YzpnOjkyMDQwMzJkMWFkOWVmYzg5MjM1ZGY2ODZjZDc0OTFiOjY6NDVlYzo4NjA1NTk3NmM3NGE4MzdiYmM0NDBmYWRkY2ZlMWE0NjI4MWQ3NDZlYjcwZDUxMWMzYmM5YzdhZjg0NTFkNGZlOnA6VDpO) **Presenters:** Dr. Sheri Madigan and colleagues Dr. Sheri Madigan is a developmental clinical psychologist and Tier II Canada Research Chair in Determinants of Child Development. Her work focuses on parent-child attachment, early adversity, and intervention.  **Organization:** University of Calgary / Madigan Lab **Contact:** Sheri Madigan ([sheri.madigan@ucalgary.ca](mailto:sheri.madigan@ucalgary.ca))  Useful websites with attachment training details:   1. [https://wwww.seasinternational.org/events/](https://protect.checkpoint.com/v2/___https://seasinternational.org/events/___.YzJ1OnN0b255YnJvb2s6YzpnOjkyMDQwMzJkMWFkOWVmYzg5MjM1ZGY2ODZjZDc0OTFiOjY6N2Y5MzpjY2I3ODA5N2JkZjlmYzRkZTFhYjdmY2VlMWU3ZWI5MTI5MzNlODk1ZDJlZWZmZDMzNTkyNWI5NDJkMDBiY2ZjOnA6VDpO) 2. [https://www.mainattachment.org/](https://protect.checkpoint.com/v2/___https://mainattachment.org/___.YzJ1OnN0b255YnJvb2s6YzpnOjkyMDQwMzJkMWFkOWVmYzg5MjM1ZGY2ODZjZDc0OTFiOjY6ZDk3YzoxODAxOGU2MWE3YTE2MDhmOTg5OTkzZjhmNTBmNTY1Y2E5YjQ2NDE0NmViMGYxY2JjZWY0OGM0NTYzODM5MDZkOnA6VDpO) 3. [https://www.ian-attachment.org.uk/](https://protect.checkpoint.com/v2/___https://www.ian-attachment.org.uk/___.YzJ1OnN0b255YnJvb2s6YzpnOjkyMDQwMzJkMWFkOWVmYzg5MjM1ZGY2ODZjZDc0OTFiOjY6YzlmYjpjNTQ2NjI3MzhhZWMyYWUyNWU2YmY0ZjBmMTBiMTYwODJhMjU1MmNmZDY5YmNjYjc0MGE1Yzg4MGQyOTU1MzhmOnA6VDpO) |
